# Screening native plants for the sustainable phytoremediation of heavy metals in industrial areas of Bangladesh

**DOI:** 10.1039/d6ra07111b

**Published:** 2026-09-29

**Authors:** Mehedi Hasan, Farjana Ashraphy, Sunjida Akhter, Shaifa Abrarin, Jannatul Tazrin, Md. Atikul Islam, Md. Ahedul Akbor, Md. Al-Amin, Md. Salim Khan, Md. Abu Bakar Siddique

**Affiliations:** a Institute of National Analytical Research and Service (INARS), Bangladesh Council of Scientific and Industrial Research (BCSIR) Dhanmondi Dhaka 1205 Bangladesh hasan.mehedi013@gmail.com sagor.bcsir@gmail.com; b Department of Chemistry, Hajee Mohammad Danesh Science and Technology University Dinajpur 5200 Bangladesh

## Abstract

Environmental pollution by heavy metals is a major concern in developing countries due to rapid and unplanned industrialization and urbanization, resulting in the contamination of soil, water, and vegetation. This poses high risks to human health and environmental systems; hence, their protection is very urgent. Here, we study the natural and environmentally friendly phytoremediation of heavy metals (*e.g.*, Pb, Cd, Cr, Mn, Fe, Co, Ni, Cu, and Zn) by identifying potential native plants collected from an industrial area (Gazipur, Bangladesh). The heavy-metal concentrations in the collected soil and plant samples were assessed using atomic absorption spectrophotometry (AAS). The potential native plants were identified by calculating the translocation factor (TF) and bioconcentration factor (BCF). The results revealed the highest TF values (∼4–8) for almost all studied metals in *Brachiaria mutica* (P-5), indicating highly efficient root-to-shoot translocation. Remarkably, *Colocasia esculenta* (P-8) and *Mimosa pudica* (P-11) showed an exceptionally high BCF (∼9) and TF (8.15), respectively, indicating high accumulation capability for Pb, while Cr was accumulated in *Stellaria media* (P-4), with a BCF of 7.45. Additionally, *Mikania micrantha* (P-3) and *Urena lobata* (P-10) exhibited high bioavailability and ease of Cd uptake through transport systems. Overall, these findings highlight the promising role of certain plant species in the phytoremediation process, offering useful insights for developing targeted remediation approaches in polluted environments and thereby enhancing environmental management for sustainable development.

## Abstract 2

Potential native plants identified for phytoremediation.

## Graphical abstract

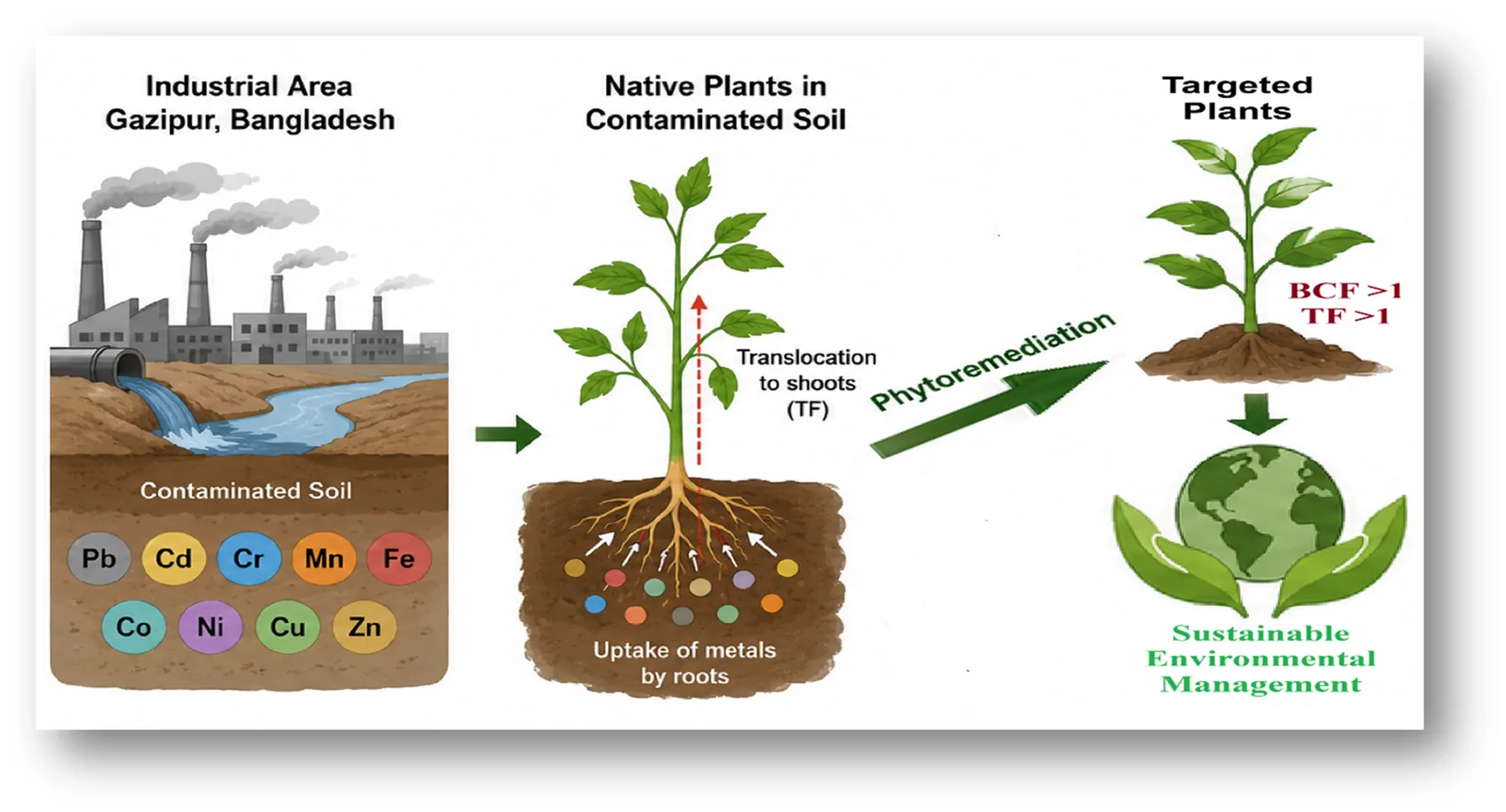

## 1 Introduction

Environmental pollution is a major concern around the world. Most of this pollution results from unplanned industrialization and urbanization, which generate a wide range of organic and inorganic pollutants. Although some organic pollutants are naturally biodegradable, inorganic pollutants, especially heavy metals, are toxic and persistent and cannot be destroyed, contaminating major environmental matrices, such as the atmosphere, water bodies, and soil.^1,2^ They can cause several adverse impacts on human health and widely degrade ecological balance.^3–5^ Developed nations continue their industrial activities by following the standard guidelines recognized by authorized organizations to minimize environmental pollution. However, developing nations like Bangladesh, one of the most rapidly industrializing nation, face challenges in carrying out such kind of activities.^6^ While industries make a substantial contribution to the economic growth and development of Bangladesh, the process of industrialization is often carried out without proper planning, resulting in continuous environmental degradation that cannot be overlooked.^7^

As an industrial region, Gazipur is a hotspot in Bangladesh with a high density of factories, including paints, textiles, electronics, chemicals, ceramics, and pharmaceuticals. Additionally, unchecked anthropogenic activities, including industrial activities, irrigation with wastewater, application of fertilizers and herbicides, and vehicular traffic, have introduced massive amounts of pollutants into the environment, likely affecting water and soil quality.^8–10^ Soil fertility, soil microorganisms, plants, animals, and human health are damaged due to the high rate of heavy metal release in the environment. Heavy metals affect soil microbial diversity by damaging DNA, inhibiting protein synthesis and cell division, and causing protein denaturation.^3^ The presence of Cd inhibits Fe(iii) reductase in roots and leads to Fe(ii) deficiency in plants, which affects photosynthetic activities.^11^ Moreover, heavy metals reduce plant biomass and cause necrosis, chlorosis, water-nutrient imbalance, and death.^12^ People are being exposed to them through inhalation and ingestion of contaminated foodstuffs. Ultimately, a noticeable proportion of residents in that particular area are suffering from various health issues like neurotoxicity and cancer diseases.^13,14^ In view of the toxic impacts of heavy metals, their removal is crucial for environmental sustainability and human health protection. However, certain uncharacterized native plants grow naturally and robustly within these contaminated zones, resulting in effective vegetation. This suggests that they are capable of accumulating toxic metals from the polluted soil and water while showing heavy-metal-resistant behavior.

This study adopts phytoremediation as a sustainable pollution control strategy, targeting heavy metal pollution in industrial and mining areas as well as urban regions. It relies on native local plants to accumulate, detoxify, and sequester toxic elements and is more environmentally friendly than traditional chemical and physical remediation approaches.^15–18^ The suite of heavy-metal remediation methods proposed in this paper can naturally maintain ecological balance and remove other pollutants in contaminated areas.^19–21^ Featuring controllable costs and environmental friendliness, it is adaptable to both urban and industrial scenarios. It can not only reduce the exposure risk of heavy metals, but also balance short-term risk mitigation and long-term ecological restoration.^22–24^ Integrating phytoremediation into urban landscapes can convert urban contaminated sites into green spaces, simultaneously improving environmental health and boosting public well-being.^25^ Existing academic research supports two efficacy-enhancing pathways: genetically modifying plants to increase their metal uptake and applying metal-chelating compounds.^26,27^ This study further verifies that mycorrhizal technology can improve the effectiveness of this remediation technology.^18^

Nowadays, phytoremediation is quite developed; however, its practical implementation and scalability in urban and industrial settings are challenging. Most of the current research is limited to laboratory experimental conditions, which cannot provide a full solution for real-world environments.^1,28,29^ No comprehensive solution has been found for how this strategy can be effectively deployed in contaminated sites and integrated into urban waste management systems. This study aims to explore plant species that can conduct suitable phytoremediation and identify the best plant-metal accumulation couple for an efficient remediation process.

This study is somewhat superior because of its naturalistic setting and real-world methodology, which are not generally applied in the traditional experiments done in controlled settings. Instead of just cultivating plants in a controlled environment, this research looks for naturally growing native plants that can stay in a polluted zone and how plant species behave directly in their natural, polluted environments. Furthermore, through the assessment of the adaptation capabilities and on-site physiological response of plants, this experiment correlates theoretical ideas associated with real-world applications, filling an important gap in current research.

Therefore, this work aims to define pollutant-selective plant species that may be suitable for contaminated sites for phytoremediation, providing a large-scale, innovative, eco-friendly, and cost-effective heavy-metal remediation process. This study may recommend the addition of phytoremediation into a wide ecological management system to provide rich guidelines and policies. Such policies are essential for effectively tackling industrial pollution challenges. The results significantly enhance urban environmental sustainability and resilience.

## 2 Materials and methods

### 2.1 Study area

This study was carried out at Noyapara, Vawal Mirzapur, Gazipur, Bangladesh (Fig. 1). There were 588 492 people living in Gazipur Upazila as of the 1991 Bangladesh census. It is situated between latitudes 23°53′ and 24°11′ north and longitudes 90°20′ and 92°30′ east. It is located approximately 41.8 kilometers north of Dhaka, the capital of Bangladesh. A large number of industries are situated in this area, such as the paint, textile, pharmaceutical, and garment industries. They produce huge amounts of waste effluents that contaminate environmental bodies, such as water, soil, and air. Due to the expansion of industrialization, deforestation has been happening day by day. Therefore, this study area is gradually losing its ecosystem, thereby impacting human daily life. Nevertheless, some native plants are growing in polluted areas, helping to keep the balance in the ecosystem. They seem to be contamination-resistant. Their existence under these circumstances suggests a high degree of tolerance to contamination and may indicate their potential ecological importance in maintaining ecosystem functions. These naturally occurring species may also possess significant phytoremediation potential.

**Fig. 1:**
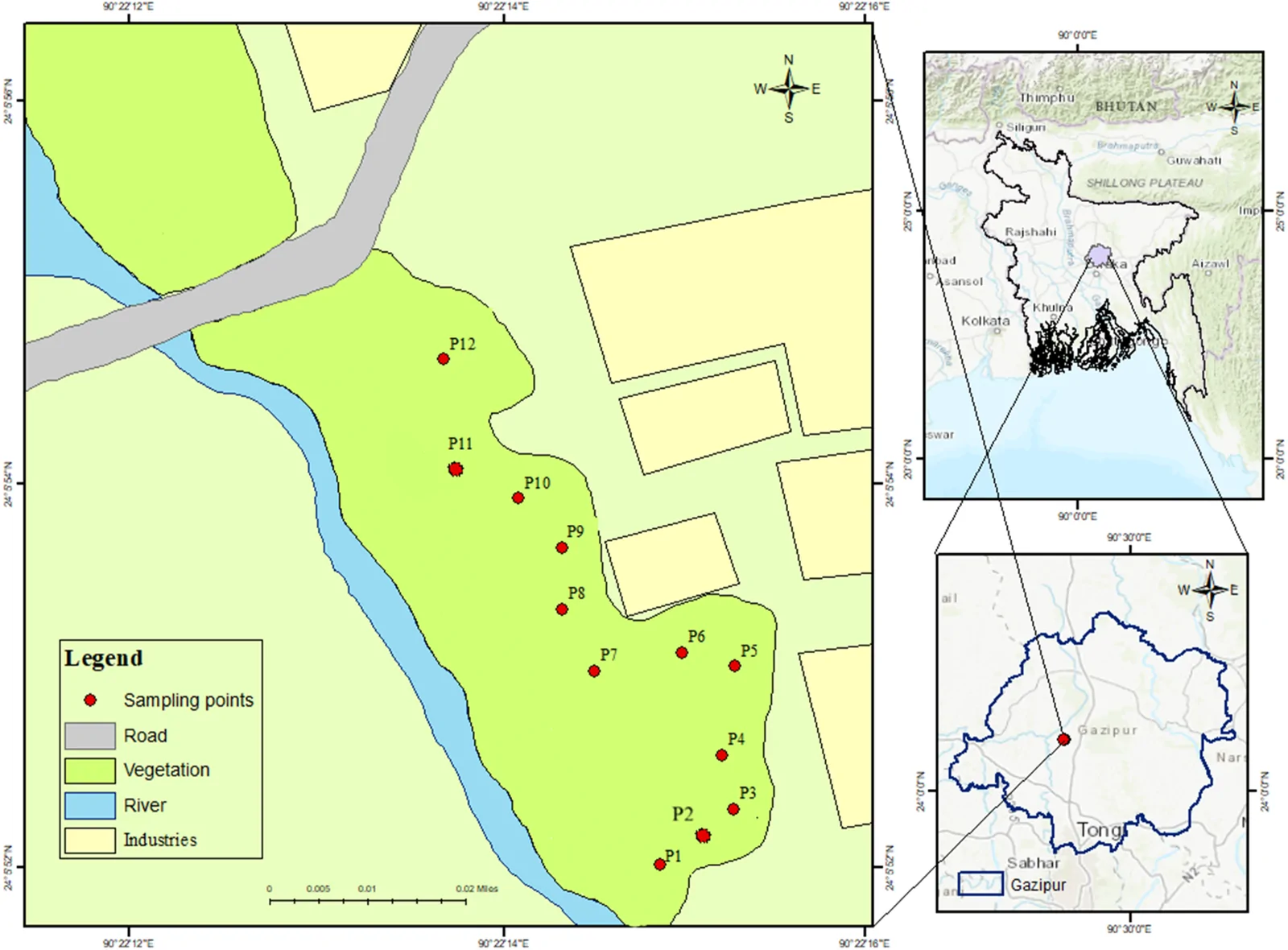
Study area at Noyapara, Vawal Mirzapur, Gazipur, Bangladesh.

### 2.2 Sample collection

In this study, 12 plant species and 12 soil composite samples were collected at twelve core sampling sites. Sampling sites were not selected based on pollution sources; rather, they were distributed across areas with varying degrees of apparent industrial influence rather than being selected to represent individual industrial sources. Soil samples were taken from the topsoil (0–25 cm) beneath the canopy of each plant until a composite sample was achieved. Three subsamples were used to make each composite sample. The raw samples were kept in polyethylene bags and transported to the laboratory. They were then stored in sealed, labeled plastic zipper bags in a refrigerator. They were selected to be investigated to determine the quantity of metals they can absorb from their surroundings. The scientific names and other identification details of the collected native plants are listed in Table 1, and the sampling points are shown in Fig. 1. The processing, preparation, and analysis of all the collected soil and plant samples were performed at the Institute of National Analytical Research and Service (INARS, an ISO/IEC 17025:2017 accredited laboratory), Bangladesh Council of Scientific and Industrial Research (BCSIR), Dhaka, Bangladesh, for nine chemical elements, *viz.,* Pb, Cd, Cr, Mn, Fe, Co, Ni, Cu, and Zn. Before sample preparation, all samples were preserved at room temperature and then prepared for elemental analysis separately.

**Table 1:** Identification of the collected plants in the industrial area in Gazipur, Bangladesh

Sampling sites	Sample ID	Scientific name	English name	Local name
ST-1	P-1	*Pennisetum purpureum*	Green napier grass	Elephant grass
ST-2	P-2	*Eichhornia crassipes*	Water hyacinth	Kachuripana
ST-3	P-3	*Mikania micrantha*	Bitter vine	Japani lota
ST-4	P-4	*Stellaria media*	Chick weed	Winter weed
ST-5	P-5	*Brachiaria mutica*	Para grass	Water grass
ST-6	P-6	*Hygrophila polysperma*	Miramar weed	Swamp weeds
ST-7	P-7	*Juncus effusus*	Common rush	Chikon ghas
ST-8	P-8	*Colocasia esculenta*	Arum spinach	Kochu
ST-9	P-9	*Clerodendrum infortunatum*	Hill glory bower	Bhat
ST-10	P-10	*Urena lobata*	Caesar weed	Bon dheros
ST-11	P-11	*Mimosa pudica*	Sensitive plant	Lojjaboti
ST-12	P-12	*Boerhavia diffusa*	Punarnava	Gondhapurno

#### 2.2.1 Chemicals and standards

Analytical-grade nitric acid (HNO_3_), perchloric acid (HClO_4_), hydrochloric acid (HCl), hydrofluoric acid (HF), and the NIST (National Institute of Standards and Technology, USA) traceable Certified Reference Materials (CRM) for all chemical elements were purchased from Fluka Analytical, Sigma-Aldrich, Germany. The chemicals and standards were utilized in their original form without performing any additional purification processes. Deionized (DI) water, with an electrical conductivity of 0.2 µS cm^−1^ and a resistivity of 18.2 MΩ cm at 25 °C, was utilized for both sample and standard preparations.

#### 2.2.2 Preparation of soil samples

After seven days of air drying at room temperature, the soil samples were oven-dried for twenty-four hours at 110 °C. Cellulosic materials and other soil contaminants were then meticulously removed using the latest procedures. A 2 mm nylon mesh screen was used to filter the materials after they were crushed and homogenized using a sterile mortar and pestle to avoid contamination. After blending and standardizing, the samples were sealed in zip-lock polythene bags and maintained at 4 °C until further analysis.^30^ Approximately 5 grams of homogenized soil samples were placed in 100 mL washed and dried beakers. The samples were digested with 10 mL of pure HNO_3_ and 5 mL of HClO_4_.^31–33^ The samples in the beakers were digested by heating them on a hot plate at 150–180 °C. They were left under a fume hood with a watch glass until nearly dry. The samples were then treated with concentrated HF (1 mL) to dissolve silicate minerals and break down the stubborn mineral matrix, allowing the complete extraction of elements from the soil's matrix.^34^ The entire procedure was repeated sequentially until a clean solution was achieved by evaporating all the degraded trace organic matter that may be present. The samples were subsequently filtered using a washed and dried 100 mL beaker. A Whatman™ qualitative 1 filter paper (125 mm diameter × 100 circles) was used for the filtration process. During filtering, the beaker containing the previous sample was rinsed several times with deionized water. After cooling to room temperature, the clear sample solutions were carefully transferred into 100 mL calibrated volumetric flasks. The flasks were rinsed multiple times with deionized water and then filled up to the mark. The solution was thoroughly mixed by shaking and then filtered into clean, labeled 200 mL plastic bottles that were not transparent. Another sample was prepared without adding soil, following the same procedure for quality control. All of the samples were meticulously kept at 4 °C in the lab for elemental analysis.^31^

#### 2.2.3 Preparation of plant samples

The fresh plant samples were meticulously washed using tap water and then purified with DI water before being left to air-dry naturally. Each plant was categorized into roots and shoots, which were then meticulously cut into tiny pieces. A calibrated digital electronic balance (Model: GR-200, A&D Company Ltd, Tokyo, Japan) was then used to measure the weight of roots and shoots in two 250 mL glass beakers, respectively, that had been cleaned and previously weighed. The roots and shoots of the plant samples in the beakers were dried in an electric oven at 110 °C for 24 hours. After cooling the beakers in a desiccator to determine the moisture content, the weight loss was then noted. After being oven-dried, samples were put in beakers and placed in an electrical Muffle furnace.^33,35^ Using a controlled temperature program that increased the temperature every 50 °C and maintained the samples at each temperature for at least 30 minutes, the temperature was progressively increased to 600 °C. The heating procedure was continued for six hours once the temperature was raised to 600 °C. After complete ashing, the root and shoot samples were digested with 10 mL of HNO_3_ and 5 mL of HClO_4_ at around 150–180 °C on a hot plate. After cooling, the samples were filtered into a 50 mL volumetric flask and stored at 4 °C for analysis.^30,33,35^ Using the same procedure as previously mentioned, a blank sample (without a sample) was also made for quality control.

### 2.3 Instrumental analysis and quality control

The nine elements—Pb, Cd, Cr, Mn, Fe, Co, Ni, Cu, and Zn—were analyzed in the soil and plant samples (roots and shoots) using atomic absorption spectrophotometry (AAS) (Model: AA240 FS, Agilent, USA), and the levels of these metals were measured. This elemental analysis was carried out using an air/acetylene flame mode. To generate a calibration curve with a linearity of at least 0.999 for every chemical element, working standard solutions were created using a different CRM for each element. By comparing the components to the calibration curve made for each element, the concentration of each component was ascertained. A hollow cathode lamp of metal-specific wavelength was used for each metal analysis under the conditions provided in Table S1. All glassware was cleaned with a 10% HNO_3_ solution for an entire night before being repeatedly cleaned with deionized water to minimize cross-contamination. As part of the upkeep of our ISO/IEC 17025:2017-accredited laboratory (INARS, BCSIR, Dhaka), the glassware (pipettes and volumetric flasks), oven, muffle furnace, and AAS equipment were calibrated from approved sources. During the analysis, the accuracy and precision of the analytical data were verified using a replication analysis of the CRM for each element. After 5 samples, the quality control sample (CRM) and method blank were analyzed in sequence. To avoid contamination from chemicals and reagents, sample blanks were generated and tested accordingly. The spike recovery in the analysis of all elements was in the range of 96–104% and was computed according to our earlier studies.^35,36^ Table S1 includes the instrumental limit of quantification (LOQ), limit of detection (LOD), calibration range, and measurement uncertainty for all examined chemical elements. All samples were measured in triplicate (relative standard deviation was less than 10%), and the mean values were used for reporting.

### 2.4 Data analysis

#### 2.4.1 Translocation factor (TF)

The movement of metals from the roots to the shoots is represented by the translocation factor (TF). It was estimated by dividing the metal concentration in the shoot by the metal concentration in the roots^16,22,37,38^ as follows:
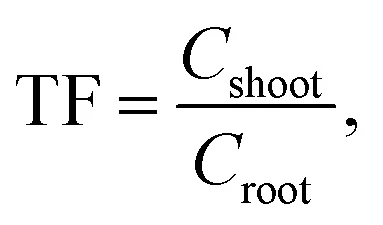
where *C*_shoot_ was the metal concentration in the shoot (mg kg^−1^), and *C*_root_ was the metal concentration in the root of the plant (mg kg^−1^).^39^ TF > 1 indicates that the plant effectively translocates trace metals from the roots to the shoots.

#### 2.4.2 Bioconcentration factor (BCF)

The bioconcentration factor (BCF) is determined by dividing the metal accumulation in the roots or whole plants by the metal content in the soil.^40–42^ It is determined as
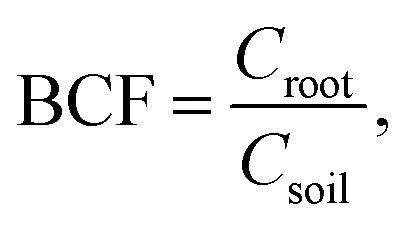
where *C*_root_ and *C*_soil_ represent the total metal concentrations in the root (mg kg^−1^) and soil (mg kg^−1^), respectively. When the BCF value is less than 1.0, 1.0–10.0, or greater than 10.0, plants are classified as excluders, accumulators, or hyperaccumulators, respectively.^39^ Moreover, phyto-stabilizers limit the bioavailability of heavy metals in the soil by immobilizing them in the roots, often with BCF and TF values less than 1, while a hyperaccumulator of heavy metals possesses a high BCF value and also transfers them effectively to its shoots (TF greater than 1).^40^ In addition, phytoextraction, as defined, is a method for eliminating soil contaminants while preserving soil structure and fertility. Nonetheless, the elevated concentrations of metals in the subterranean tissues and the restricted transfer of these metals to the above-ground tissues in the examined plants suggest that these species possess a well-controlled capacity to take up and transfer metals, even in extremely contaminated settings.^43^

### 2.5 Statistical analysis

For this study, data were collected and stored using Excel 2019. A principal component analysis (PCA) was performed to determine the relationships between plant species according to their BCF and TF values for each metal. The data were normalized by *z*-score, subtracting the mean and dividing by the standard deviation. The metals (Pb, Cd, Cr, Mn, Fe, Co, Ni, Cu and Zn) were considered as variables. BCF, TF and PCA were calculated using Origin Software (version Pro 2024).

## 3 Results and discussion

### 3.1 Characteristics of plant species and levels of metals in soil and plants

As seen in Table 2, the plants examined accumulated significant quantities of some potentially toxic elements, indicating their ability to absorb and retain contaminants like live nutrients from the environment around them. Fe and Mn were the most abundant elements due to their biological importance. However, the accumulation of Pb, Cd, Cr, Co, Ni, Cu, and Zn needs special attention due to their environmental contamination, non-biodegradability, and potential ecological and human health risks, indicating the impacts of unplanned industrial activities.^44,45^ Lead (Pb) concentrations varied from 5.7 to 98.5 mg kg^−1^, with a mean value of 29.8 mg kg^−1^. The highest concentration of Pb was found in *Mimosa pudica* (P-11), followed by *Colocasia esculenta*, P-8 (92.2 mg kg^−1^). These concentrations are much higher than the normal Pb concentration (generally 0.1–10 mg kg^−1^), indicating apparent Pb enrichment. This may be attributed to the extensive roots of *Mimosa pudica,* which can interact with Pb retained in the topsoil. Root exudates, such as organic acids, amino acids and phenolic compounds, can alter Pb solubility and bioavailability.^46,47^ The highest concentrations of the most toxic trace metals, cadmium (4.52 mg kg^−1^) and nickel (112.5 mg kg^−1^), were found in *Mikania micrantha* (P-3). Therefore, the high levels of Cd and Ni in many of the samples may be indicative of the presence of Ni-rich substrates or anthropogenic contamination and may be a reflection of metal stress. The highest accumulation of both Cd and Ni was in *Mikania micrantha*, which may be partly related to its large root system, rapid biomass production, and efficient uptake of divalent metal ions through broad-specificity transport proteins.^48,49^ Extensive root systems can increase root–soil contact and interaction with available metal species, potentially favoring metal accumulation in roots. Following uptake, membrane transport proteins, such as natural resistance-associated macrophage protein (NRAMP), zinc-regulated transporter (ZIP), and heavy metal ATPases (HMA) families, may partially contribute to the acquisition, intracellular trafficking, and xylem loading of metal ions. Metals can subsequently be complexed by phytochelatins, metallothioneins, and organic acids and sequestered in vacuoles, thereby reducing their toxicity.^50^ The maximum content of chromium (61.1 mg kg^−1^), copper (239.0 mg kg^−1^), and zinc (2339.0 mg kg^−1^) was documented in *Stellaria media* (P-4). Values are higher than concentrations usually reported in uncontaminated vegetation, indicating a high availability of Cr and Cu in the sampling area.^51^ The observed concentrations indicate a high capacity for accumulation, but they do not seem to negatively affect photosynthetic efficiency and cellular metabolism, which may suggest a high resistance of the plant species to metals. In general, the P-3, P-4, P-8, and P-11 plant species consistently showed high levels of several toxic metals, indicating localized contamination hotspots or increased metal bioavailability. The accumulation of several metals in these plants suggests that they possibly can be good bioindicators of pollution in the environment and also may have potential for phytoremediation, depending on their biomass and translocation capacity. The concentrations of potentially toxic metals in the investigated soils exhibited moderate spatial variation among the sampling sites (Table 3). Overall, the average concentrations were in the following order: Cu > Ni > Zn > Pb > Co > Cr > Cd. Among the toxic metals, Cu showed the highest mean concentration (42.5 mg kg^−1^), followed by Ni (30.1 mg kg^−1^), Zn (19.6 mg kg^−1^), Pb (11.3 mg kg^−1^), Cr (5.72 mg kg^−1^), and Cd (0.16 mg kg^−1^).

**Table 2:** Concentration of total heavy metals (mg kg^−1^) in plant parts (roots and shoots) collected from an industrial area in Gazipur and regulatory standards

Plant species	Sample ID	Total heavy-metal concentration (mg kg^−1^) in plant parts								
		Pb	Cd	Cr	Fe	Mn	Co	Ni	Cu	Zn
*P. purpureum*	P-1	12.9	0.19	26.5	15 894.8	860.8	7.60	31.0	57.1	441.6
*E. crassipes*	P-2	24.9	0.42	52.8	18 120.0	5317.1	8.08	46.8	86.6	354.2
*M. micrantha*	P-3	22.2	4.52	16.0	3292.0	3827.4	0.14	112.5	191.5	1350.1
*S. media*	P-4	30.5	0.55	61.1	31 856.8	8137.2	19.6	77.6	239.0	2339.0
*B. mutica*	P-5	26.5	0.37	42.6	18 122.0	507.7	6.17	41.1	160.8	1013.5
*H. polysperma*	P-6	14.5	0.42	12.0	10 572.4	11 572.4	31.7	45.7	88.1	1350.5
*J. effusus*	P-7	12.6	0.13	14.3	18 034.5	3335.0	8.72	25.4	25.0	228.6
*C. esculenta*	P-8	92.2	1.59	40.8	7916.0	2999.6	20.4	52.4	211.5	400.5
*C. infortunatum*	P-9	5.7	0.51	28.4	2988.7	3385.1	3.24	22.3	52.0	349.0
*U. lobata*	P-10	7.0	3.08	2.9	1573.5	7374.2	5.61	19.3	44.8	675.0
*M. pudica*	P-11	98.5	0.04	49.5	6328.7	1000.7	3.09	26.6	19.8	224.3
*B. diffusa*	P-12	9.9	0.16	8.1	8563.5	7040.7	11.1	28.7	84.2	525.7
Max.		98.5	4.5	61.1	31 856.8	11 572.4	31.7	112.5	239.0	2339.0
Min.		5.7	0.0	2.9	1573.5	507.7	0.1	19.3	19.8	224.3
Average		29.8	1.0	29.6	11 938.6	4613.2	10.5	44.1	105.0	771.0
FAO/WHO		5.0	0.3	5.0	450.0			0.1		60.0

**Table 3:** Concentration of heavy metals (mg kg^−1^) in soils collected from beneath the canopy of each plant in the industrial area in Gazipur

Sample ID	Concentration of heavy metals (mg kg^−1^) in soils								
	Pb	Cd	Cr	Fe	Mn	Co	Ni	Cu	Zn
S-1	10.0	0.16	7.49	25 431.3	308.8	8.76	32.1	76.6	23.4
S-2	11.9	0.25	7.66	23 861.3	181.3	7.64	30.6	32.5	17.2
S-3	13.6	0.21	7.65	24 251.3	150.0	8.60	29.3	48.7	15.2
S-4	11.6	0.10	3.05	25 078.8	355.0	9.21	22.4	18.3	16.9
S-5	7.12	0.13	4.15	21 603.8	262.5	7.81	22.4	27.6	17.1
S-6	8.25	0.30	6.80	29 233.8	481.3	11.6	36.8	77.1	17.8
S-7	7.50	0.18	6.50	24 227.5	336.3	8.01	29.7	54.7	22.5
S-8	10.1	0.20	6.70	22 040.0	263.8	7.24	27.8	60.9	25.2
S-9	14.6	0.05	5.22	37 711.3	3860.0	18.5	45.2	28.5	21.5
S-10	14.3	0.14	3.50	28 933.8	3405.0	10.2	23.4	15.4	22.8
S-11	14.9	0.06	4.20	29 402.5	3026.3	8.56	31.0	27.2	15.9
S-12	11.3	0.16	5.72	26 525.0	1148.2	9.65	30.1	42.5	19.6
Max.	14.9	0.30	7.66	37 711.3	3860.0	18.5	45.2	77.1	25.2
Min.	7.12	0.05	3.05	21 603.8	150.0	7.24	22.4	15.4	15.2
Average	11.3	0.16	5.72	26 525.0	1148.2	9.65	30.1	42.5	19.6
FAO	100	3	100	—	—	—	50	4.5	300

Although essential elements (Fe and Mn) were present at much higher concentrations, the distribution of toxic metals provided a better indication of anthropogenic contamination and ecological risk. Overall, the concentrations of toxic metals in the soils were well below the threshold values established by international soil-quality guidelines, indicating that the soils were not heavily contaminated.^52^ The contents of potentially toxic metals revealed moderate spatial variability in the study areas (Table 3).

Nevertheless, the data presented in this study, regarding the presence of an appreciable amount of Fe, Ni, and Cu, along with the high content of metals in the corresponding plant samples, suggested metal bioavailability, rather than their total content in the soils. Soil properties, including pH, organic matter content, CEC, and metal speciation, are known to influence metal mobility and bioavailability, control their uptake by plants, and may therefore contribute to the observed differences. However, these parameters were not determined in the present study, and their specific contributions cannot be directly established. Further studies incorporating detailed soil physicochemical characterizations and metal speciation analysis are needed to clarify the mechanisms governing metal uptake. The relatively high metal concentrations in plant tissues compared with the corresponding soils in this study may also suggest that the studied plants may demonstrate high efficiency in metal uptake, which could potentially be used in phytoremediation efforts.^31,37,53–55^

### 3.2 Bioconcentration factor (BCF) and translocation factor (TF) of heavy metals in the investigated plant species

The bioconcentration factor (BCF) and translocation factor (TF) are two crucial indices for determining the phytoextraction potential of a particular plant species. The former implies the ability of a plant species to accumulate metals in its tissues from the underlying soil, while the latter indicates how much of the absorbed metal is translocated from the roots to the plant's shoots. For phytoextraction, the most suitable plant species are those with both BCF and TF higher than 1, whereas for phytostabilization, plants with a high BCF and a low TF are the most appropriate.^56–58^ As shown in Fig. 2, the results of BCF and TF are presented for each metal, and the horizontal ordinate numbers represent different kinds of plants. It can be seen that both indicators varied significantly, which may denote differences in metal uptake, sequestration, and transportation mechanisms. For instance, the majority of species had BCF values below 1 for Pb, which indicated a poor ability to accumulate in plant roots. This was explained by the poor Pb bioavailability in the soil due to its strong adsorption on soil particles and root cells, thereby limiting its transportation in plant tissues.^46,47^ However, *Colocasia esculenta* (P-8) was found to exhibit an exceptionally high BCF (∼9), which may suggest a substantial ability to accumulate Pb, allowing it to possibly serve as a key species for the phytoremediation of Pb around battery industries due to its wide root system and relative tolerance to heavy metals.^59^ This behavior was associated with strong Pb retention at the root–soil interface and binding of Pb to negatively charged functional groups of organic acids in root tissues. A major fraction of metals entering roots may be retained in the cell wall and apoplast. Functional groups in pectin, cellulose, and hemicellulose—particularly carboxylate (–COO^−^), hydroxyl (–OH), and phenolic groups—can bind metal ions as follows.^60,61^R–COO^−^ + Pb^2+^ → R–COOPb^+^.

**Fig. 2:**
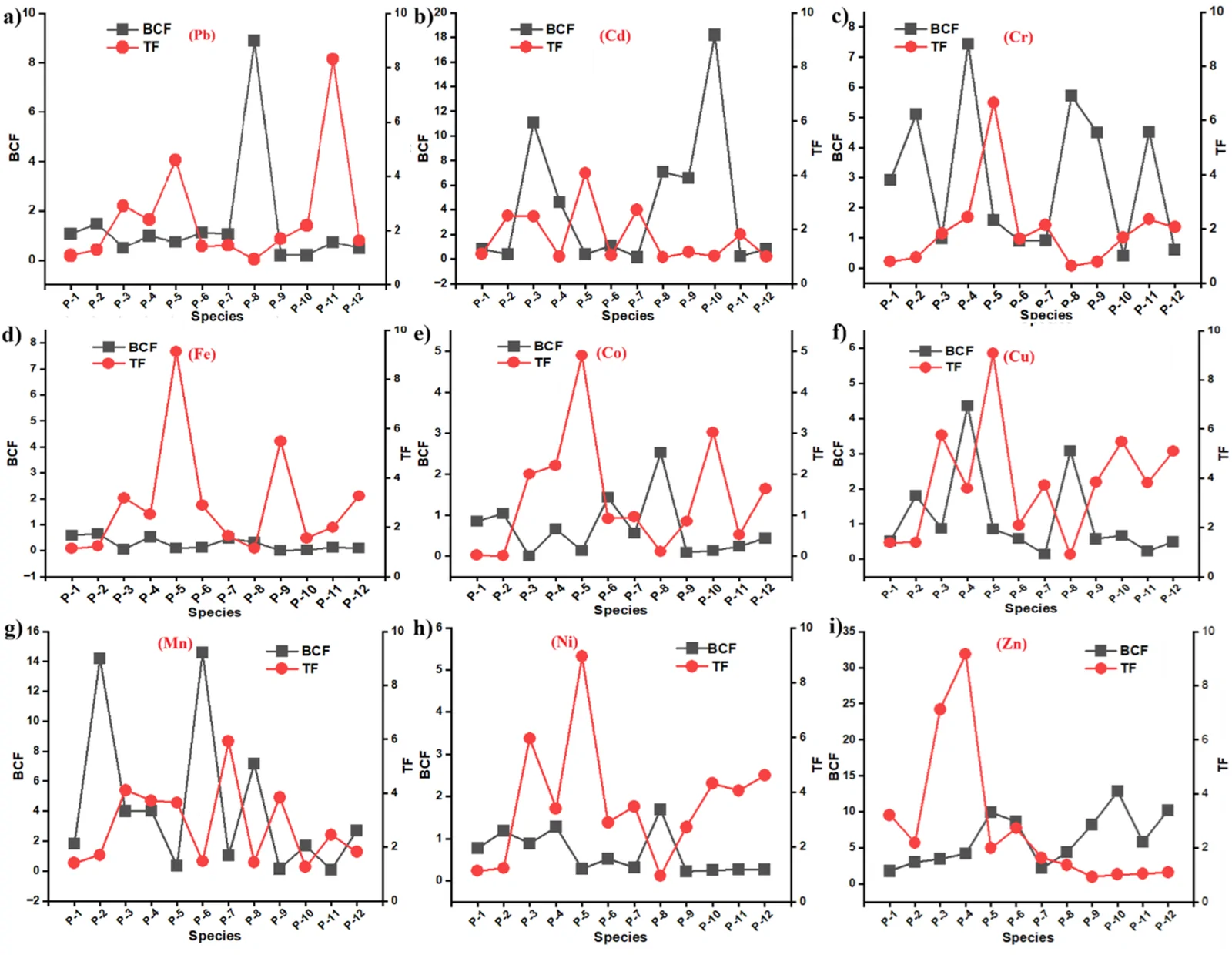
BCF and TF values of metals in the investigated plant species: (a) Pb, (b) Cd, (c) Cr, (d) Fe, (e) Co, (f) Cu, (g) Mn, (h) Ni, and (i) Zn (the horizontal ordinate numbers represent different kinds of plants as follows: P-1: *Pennisetum purpureum*; P-2: *Eichhornia crassipes*; P-3: *Mikania micrantha*; P-4: *Stellaria media*; P-5: *Brachiaria mutica*; P-6: *Hygrophila polysperma;* P-7: *Juncus effusus*; P-8: *Colocasia esculenta*; P-9: *Clerodendrum infortunatum*; P-10: *Urena lobata*; P-11: *Mimosa pudica*; and P-12: *Boerhavia diffusa*).

In comparison, the *M. pudica* (P-11) species exhibited the highest TF value (8.15) among all the studied plants despite its low BCF value. This suggested that the species had a good ability to translocate Pb from the roots to the shoots after its uptake. Based on the high TF value, it can be suggested that this species may have a better phytoextraction potential compared to other plants with high BCF, which do not efficiently translocate the metal to the shoots.^44,62^ Another possible explanation could be related to the adaptation mechanisms through which plants can tolerate high metal concentrations: sequestering it into the vacuoles of root cells or xylem vessels or complexing it with organic acids, phytochelatins, and other ligands.^63^ These might also comprise precipitates of carbonate, sulfate, and phosphate, which are later immobilized in the intracellular space (symplastic portions, such as vacuoles) or the outside of the cell (apoplastic cellular walls). The xylem stream in the root symplasm may absorb the metal ions that are probably held inside the vacuoles as they travel to the stele.^64^ The metal ions are then delivered to the shoots by the xylem vessels. In order to prevent the buildup of free metal ions in the cytosol, the apoplast or symplast, which stores the ions in extracellular sections (cell walls) or the plant vacuole, permits them to flow through and disperse throughout the leaves.^65^ Cadmium (Cd) demonstrated relatively high BCF values (∼3.5) for most species, with *M. micrantha* (P-3) and *C. infortunatum* (P-9) showing comparatively greater Cd accumulation. This pattern may reflect the ability of Cd^2+^ to enter plant roots through transport pathways involved in the uptake of essential divalent cations, including Zn^2+^, Fe^2+^, and Ca^2+^. However, Cd's TF varied considerably between species, with the highest being *B. mutica* (P-5). Plausible reasons may be significant interspecific differences in Cd chelation, vacuole sequestration, and xylem loading, which may be responsible for the variation in root-to-shoot translocation and phytoextraction potential.^66,67^ For chromium, a high affinity for accumulation was also observed among the tested species, with *S. media* (P-4) demonstrating the highest BCF of 7.45, while *B. mutica* (P-5) showed the highest TF of 5.49. Generally, Cr is considered a hard metal to translocate in plants due to its moderate adsorption on roots and limited xylem entrance, which decreases its transfer through the vascular system. Thus, the observed TF values can be used as a basis for understanding the peculiarities of Cr physiology in different plant species.^43,44,62,68^ Copper and manganese demonstrated different trends in their bioaccumulation. Copper revealed moderate BCF values, reaching a maximum level of 4.36 in *Stellaria media* (P-4), while the translocation factor was the highest (5.86) in *Brachiaria mutica* (P-5). As an essential micronutrient, manganese presented notably high BCF values, which were even higher than those of most metals. Indeed, there were specimens presenting BCF values of 14.2 and 14.6 for *Eichhornia crassipes* (P-2) and *Hygrophila polysperma* (P-6), respectively. However, significant variations in TF suggested Mn homeostasis mechanisms that varied considerably between species.^69,70^

Cobalt revealed moderately low BCF values in all plant species under consideration except the other three species mentioned above, while iron demonstrated low BCF values (<1.0) for all species and was consistently translocated to a considerable extent, hinting at a certain level of tolerance to metal toxicity.^70^ Indeed, the observed patterns of accumulation might be related to the homeostasis mechanisms for Co and Fe, limited membrane transporters for these metals, or their low bioavailability.^71–73^ However, both metals could be translocated to aerial parts *via* xylem, in the form of metal–ligand complexes, which resulted in moderate or high TF values.^69,70^

Among metals, zinc demonstrated the most remarkable translocation rates, with TF values of 24.21 and ∼31.87 in *Mikania micrantha* (P-3) and *Stellaria media* (P-4), respectively, while the BCF value increased steadily from moderate levels and reached its peak in *Urena lobata* (P-10) at 14.91. Being an essential micronutrient with a relatively high degree of mobility, Zn can be efficiently translocated *via* xylem and phloem, resulting in extraordinarily high TF values compared to other metals. Thus, there is a possibility of using some of the studied species for the phytoextraction of zinc. Surprisingly, *Brachiaria mutica* (P-5) demonstrated the maximum TF values (∼4–8) for Pb, Cd, Cr, Fe, Co, Cu, and Ni. This indicated that, regardless of the moderate levels of bioaccumulation, they could be efficiently translocated to above-ground plant parts through the xylem and possibly contribute to phytoextraction due to the ease of removing the aerial parts of the plant from the site. Therefore, the high TF values can indicate the presence of efficient long-distance transport mechanisms for metals, which can be useful for phytoextraction.^43,44^ Overall, these processes provide plausible chemical and physiological explanations for the variation in BCF and TF observed among the investigated species. In particular, high BCF accompanied by low TF may indicate preferential root retention and sequestration, whereas simultaneous high BCF and TF may indicate efficient uptake and root-to-shoot transport. However, because soil pH, organic matter, CEC, metal speciation, root exudates, transporter expression, and subcellular metal distribution were not measured, these mechanisms should be considered plausible interpretations rather than experimentally demonstrated causes of the observed accumulation patterns.

### 3.3 Principal component analysis (PCA)

Principal component analysis (PCA) was applied to explore the relationships between the studied species based on the BCF and TF values of the selected metals. Both BCF and TF were analyzed together. The analysis revealed five (5) principal components with eigenvalues higher than 1 (Fig. 3a). However, the contribution of the other components was lower, and only the first two principal components (PC1 and PC2) were used to plot the graphical visualization (Fig. 3b). This was because together they explained the largest proportion of the total variance (58.11%) and adequately represented the multivariate relationships among the variables. Apparently, 50–60% of variance is considered acceptable for PCA and is sufficient for the further interpretation of results.^74^

**Fig. 3:**
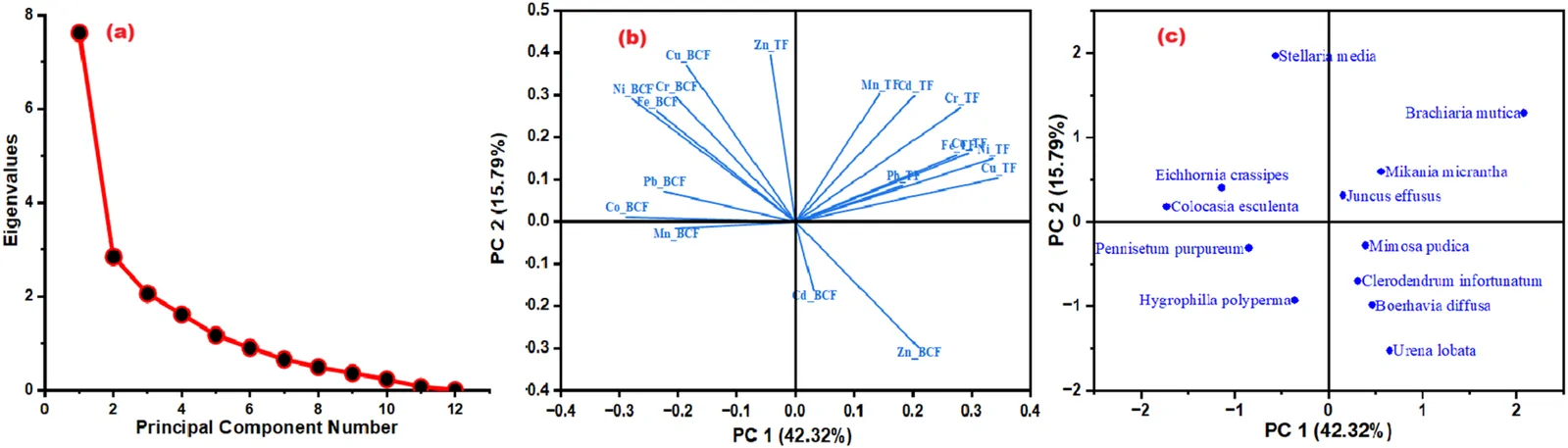
Principal component analysis (PCA) of metal accumulation in the investigated plant species: (a) scree plot, (b) loading plot, and (c) score plot.

The PCA score plot (Fig. 3c) revealed that the investigated plant species could be grouped based on their ability to accumulate and translocate heavy metals. The species that were close to each other showed similar responses to heavy metal contamination, whereas the species that were far from each other showed an opposite trend. Specifically, *Brachiaria mutica* (P-5) had the highest score on PC1 (2.085) among all species, implying that this species had the highest value on the variables that had positive loadings on PC1.

As can be seen in Fig. 3b, Cu_TF, Ni_TF, Co_TF, Cr_TF, Fe_TF, Mn_TF, and Cd_TF were positively loaded on PC1, suggesting that *B. mutica* had a high level of root-to-shoot translocation of these heavy metals. Therefore, this species could be considered a good candidate for phytoextraction due to its high TF of multiple heavy metals. Another species, *Mikania micrantha* (P-3), was found to be in the same quadrant as *B. mutica*. Hence, it also had positive scores for PC1 and PC2, suggesting that it had positive values for most of the variables positively loaded on these two components. Compared to *B. mutica, M. micrantha* had a lower score for PC1, implying that its level of TF was lower than that of B. mutica. On the contrary, *Colocasia esculenta* (P-8) (−1.721, 0.179), *Eichhornia crassipes* (P-2) (−1.137, 0.401), and *Stellaria media* (P-4) (−0.567, 1.966) were found to be in the lower left quadrant, suggesting that these species had negative scores for PC1, which was positively loaded on Pb_BCF, Cr_BCF, Fe_BCF, Mn_BCF, Co_BCF, Ni_BCF, Cu_BCF. This indicated that these species were associated with metal accumulation rather than metal translocation. This suggested that these species could be suitable candidates for phytostabilization since they were capable of accumulating heavy metals in their roots. The species located in the lower right quadrant were *Urena lobata* (P-10), *Boerhavia diffusa* (P-12), *Clerodendrum infortunatum* (P-9), and *Mimosa pudica* (P-11). These species mostly had positive scores for PC1 but negative scores for PC2, suggesting that they had moderately high TF for most of the heavy metals but were low accumulators of heavy metals. This was further supported by their proximity to each other on the score plot. Specifically, since PC2 was positively loaded on Zn_TF, Cu_BCF, Cd_TF, and Mn_TF, these species had relatively lower TF and lower BCF than P-3 and P-5. Two other species, *Pennisetum purpureum* (P-1) and *Hygrophila polysperma* (P-6), were located in the lower left quadrant, suggesting that these species had lower TF than most of the other species. *Juncus effuses* (P-7) was located close to the origin of the PCA score plot, implying that this species had an intermediate level of TF for most of the heavy metals.

In summary, the analysis of the score plots and the loading plot revealed that the evaluated plant species varied greatly in their abilities to accumulate and translocate heavy metals. Species located in the left upper quadrant (*B. mutica* (P-5) and *M. micrantha* (P-3)) were associated with high TF of heavy metals and could be good candidates for phytoextraction. Species that were located in the lower left quadrant (*C. esculenta* (P-8), *E. crassipes* (P-2), *S. media* (P-4), *P. purpureum* (P-1), and *H. polysperma* (P-6)) were associated with high BCF of most heavy metals and could be good candidates for phytostabilization.

## 4 Comparison with other studies

Table 4 shows some previous Bangladeshi studies demonstrating phytoremediation potential in several industrially affected environments with varied scopes. Hasan *et al.* (2021) focused specifically on Cr contamination associated with the Savar tannery industry and identified *X. strumarium* as a promising native Cr accumulator based on its BCF and TF values.^37^ Juel *et al.* (2021) experimentally evaluated two plants in Hazaribagh tannery sludge and reported contrasting accumulation patterns between *B. juncea* and *P. purpureum.*^75^ Riza and Hoque (2021) assessed the capacity of *B. pinnatum* to accumulate multiple metals in textile industrial soil.^76^ The present study extends this body of evidence through a comparative field assessment of 12 naturally growing native plant species against nine potentially toxic elements within the same industrially impacted landscape. Rather than concentrating on a single contaminant and a single plant species, the study combines multi-metal accumulation analysis with BCF, TF, and PCA, which may contribute to resolving species-specific accumulation and translocation behavior. The resulting classification of plants according to their potential for phytoextraction *versus* phytostabilization provides a more integrated basis for selecting locally adapted plant communities for heavy-metal management under the environmental conditions of Mirzapur, Gazipur.

**Table 4:** Comparison of phytoremediation studies conducted in Bangladesh

Location	Plant species	Target metals	References
Savar tannery industrial estate	*Eichhornia crassipes*, *Xanthium strumarium* L., *Cynodon dactylon*, *Croton bonplandianum*	Cr	37
Hazaribagh tannery area	*Pennisetum purpureum*, *Brassica juncea*	Cr, Pb, Cu, Zn	75
Kaliakair, Gazipur	*Bryophyllum pinnatum*	Cu, Ni, Cr, Pb, Cd	76
Noyapara, Vawal Mirzapur, Gazipur	12 naturally growing plants mentioned in Table 1	Pb, Cd, Cr, Mn, Fe, Co, Ni, Cu, Zn	Present study

## 5 Conclusion

This study demonstrated that the investigated native plant species growing in industrially polluted areas differed substantially in their ability to accumulate and translocate heavy metals, indicating species- and metal-dependent phytoremediation potential. Several species, especially *Colocasia esculenta* (P-8) and *Mimosa pudica* (P-11), showed favorable BCF and TF characteristics, respectively, and may therefore serve as potential candidates for phytoremediation. However, their practical remediation capacity requires further validation through controlled experiments, seasonal monitoring, and biomass-based assessments.

Based on the observed accumulation and translocation patterns, this potential strategy could be considered for contaminated industrial areas in Bangladesh. Species with high BCF and relatively low TF may be preferentially planted in highly contaminated zones, where metal retention in the root zone is desirable, whereas species exhibiting both high BCF and TF could be evaluated in harvestable planting zones for potential phytoextraction. A multilayer arrangement of herbaceous and large native vegetation along industrial boundaries could additionally contribute to reducing soil erosion and dispersal of metal-containing particulates. However, planting density, species combinations, harvesting frequency, and long-term metal removal capacity should be established through pilot-scale field trials.

Therefore, the findings provide a basis for selecting locally occurring plant species for further phytoremediation research and for developing site-specific green-belt strategies in the industrial regions of Bangladesh. Future studies should evaluate individual and mixed-species planting systems across seasons and industries, together with plant biomass, metal uptake rates, soil physicochemical properties, and metal speciation. Such information would allow the total metal-removal capacity and long-term effectiveness of these plants to be quantitatively assessed and would support the development of practical phytoremediation systems suited to Bangladesh's tropical environment.

## Author contributions

Mehedi Hasan: conceptualization, methodology, investigation, formal analysis, data curation, visualization, writing – original draft, writing – review & editing. Farjana Ashraphy: methodology, investigation, visualization, writing – original draft. Sunjida Akhter, Saifa Abrarin, and Jannatul Tazrin: methodology, investigation, formal analysis, data curation. Dr Md. Atikul Islam: methodology, investigation, writing – review & editing, supervision. Dr Md. Ahedul Akbor: investigation, formal analysis, data curation, writing – review & editing, supervision. Md. Al-Amin: investigation, methodology. Dr Md. Salim Khan: writing – review & editing. Md. Abu Bakar Siddique: conceptualization, methodology, investigation, formal analysis, data curation, visualization, writing – original draft, writing – review & editing, supervision.

## Conflicts of interest

The authors declare that they have no known competing financial interests or personal relationships that could have appeared to influence the work reported in this paper.

## Supplementary Material

RA-OLF-D6RA07111B-s001

## Data Availability

Data will be made available on request. Supplementary information (SI) is available. See DOI: https://doi.org/10.1039/d6ra07111b.
