# Supplementary material for: Screening native plants for the sustainable phytoremediation of heavy metals in industrial areas of Bangladesh
Source: RSC Adv. 2026 Sep 29;16(45):58219–31. doi: 10.1039/d6ra07111b (PMC13621246; doi:10.1039/d6ra07111b)
Supplement: Supplementary file 1 [file RA-016-D6RA07111B-s001.pdf]

Supplementary Information (SI) for RSC Advances.
This journal is © The Royal Society of Chemistry

Table S1

Experimental parameters of the element-specific hollow cathode lamp used in the AAS instrument during the measurements of the samples along with spike recovery(%), limit of quantification (LOQ), the limit of detection (LOD), calibration range, and measurement uncertainty for all the analyzed chemical elements.

Elements	Experimental parameters of hollow cathode lamp			LOQ (μg/L)	LOD (μg/L)	Calibration range (μg/L)	Spike recovery (%)	Measurement uncertainty(±) (K=2)
	Lamp current (mA)	Wavelength (nm)	Slit width (nm)					
Fe	5.0	248.3	0.2	100	67.7	100-2000	100.3	8%
Mn	5.0	279.5	0.2	50	3.1	50-1000	101	7%
Zn	5.0	213.9	1.0	50	7.5	50-1000	98.9	11.6%
Cu	4.0	324.8	0.5	100	68.2	100-2000	99.6	7.2%
Co	7.0	240.7	0.2	100	62.1	100-2000	100.4	8%
Pb	10.0	217.0	1.0	100	65.0	100-2000	99.9	6.5%
Ni	4.0	232.0	0.2	100	68.5	100-2000	100.6	8.4%
Cd	4.0	228.8	0.5	25	0.3	25-400	99.2	7%
Cr	7.0	357.9	0.2	100	70	100-2000	100.2	8%
